# Study on the Microclimate Effect of Water Body Layout Factors on Campus Squares

**DOI:** 10.3390/ijerph192214846

**Published:** 2022-11-11

**Authors:** Han Xu, Xinya Lin, Ying Lin, Guorui Zheng, Jianwen Dong, Minhua Wang

**Affiliations:** 1 College of Landscape Architecture, Fujian Agriculture and Forestry University, 15 Shangxiadian Rd., Fuzhou 350002, China; 2 Engineering Research Center for Forest Park of National Forestry and Grassland Administration, 63 Xiyuangong Rd., Fuzhou 350002, China

**Keywords:** water body layout factors, campus square, microclimate effect, ENVI-met

## Abstract

Quantifying the water layout factors in a campus square helps to lay out water bodies more scientifically and utilize the microclimate effect to alleviate the heat and humidity of campus squares in summer. The West Gate Square of Fujian Agriculture and Forestry University in China has been used as the primary theoretical model, and the landscape pattern index from landscape ecology has been used to quantify the scale, shape, and dispersion of water bodies. Consider the typical weather, the summer solstice, as the experiment time. The relationship between the water body layout factors and cooling effect, the humidification effect, and the wind speed is clarified from both temporal and spatial perspectives. The data were analyzed with ENVI-met and Arcgis software. Then, the optimum campus square water body layout mode was concluded. The results show that: (1) The scale, dispersion, and shape of the water body has a significant effect on the campus temperature and humidity, while the effect on wind speed is not significant. (2) From the cooling and humidifying effect, the ranking of the regulating ability of the water body layout factors is scale > shape > dispersion; the ranking of the influence range is shape > scale > dispersion. (3) When the boundary of the square is determined, the optimum water body layout mode is that the water body area accounts for 36% of the total square area. The shape of the water body is concentrated and not dispersed square. When the water body layout is determined, the optimum layout mode of the boundary is length:width = 1:2.

## 1. Introduction

Landscape microclimate has become a research hotspot with the deterioration of climate conditions, and how to create a healthy and comfortable campus environment has become an issue for scholars. Some scholars focus on the relationship between the green space of the campus, plant community characteristics and the microclimate [1,2]. Tobi and Ling Zhang [3,4] found that “tree arrangement, LAI, range of tree-crown and the height of trees” can influence the effect of vegetation on the thermal environment and ventilation. Sijia Wu [5] and Mallen [6] et al. found that the campus had several types of substrates: vegetation, water, and hard paved areas. The cooling capacity of different substrates was trees > grass > water > hard paved areas. Yi Huang studied the microclimate effects of the subsurface, vegetation, buildings, and road orientation with an example of the campus square [7], but ignored the effect of campus water bodies on the microclimate environment. Compared with other landscape factors, the structure of water bodies is complex and difficult to change once established [8]. Therefore, the design of water bodies for campus squares needs to be more rigorous.

It has been shown that water bodies have a significant impact on the microclimate [9]. On the one hand, water bodies have different degrees of environmental impact due to their physical structure. Firstly, a unique microclimate is typically produced above water bodies due to the properties of high heat capacity, high latent heat of evaporation, and low reflectivity of the water surface. It has regional, seasonal, and diurnal effects on the air temperature, relative humidity, and wind speed of the surrounding environment [10]. Secondly, wind speed and direction change the extent and the impact of the water body microclimate [11]. As an open space, the overall roughness of the water body is smaller than the area covered by the building. This can form wind channels and increase the wind speed to improve the natural ventilation of the campus. Influenced by the dominant wind direction, the degree of influence and propagation of the cooling and humidifying effect in the downwind water body area is greater [11,12,13]. On the other hand, the state [14], size [15], morphology [16], and dispersion [17] of water bodies are closely related to the microclimate effect. Relevant water body microclimate research has shown that different layouts of water bodies create different urban microclimate effects [18]. Xu evaluated the effects of urban water bodies during hot weather with temperatures above 35 °C. The results showed that the increasing water body area decreases the heat index in hot summer and the microclimate environment in coastal areas is significantly improved [19]. Jin, H found that the larger the water body scale, the wider the microclimate improvement [14]. Based on that, Lei Zhang tried to explore the effect of water depth on the microclimate. The effect on temperature and humidity remained the same when the depth of water exceeded 4 m [20], indicating that the depth of the water body has little effect on the microclimate. Xuan found that the cooling effect of dynamic water bodies is better than static water bodies [21]. When the fountain is open, the temperature difference between the upper and lower wind direction is about 1.3 °C, and the relative humidity difference is about 7.5%. When the fountain is closed, the difference in temperature between the upper and lower wind direction is about 1 °C; the relative humidity difference is about 2% [22]. Besides the area of water bodies, the single water body shape, and the combination of multiple water bodies in different ways can also produce different microclimate effects. Offerle found that the shape and dispersion of water bodies play a decisive role in the differences between surface and air temperatures [23]. Sun argued that the range and extent of water body cooling effects depend on the size and shape of the water body, and the characteristics of the surrounding area [24]. Currently, relevant research generally focuses on the microclimate change caused by water bodies in actual measurement and analysis [25,26], and the systematic research on the microclimate effect of different water body layout factors is lacking. Therefore, it is necessary to quantify the layout factors of water bodies and analyze their microclimate effects to provide a theoretical basis and a data reference for improving the microclimate environment of campus squares and to enrich the research on the campus microclimate.

Against the background of resource conservation, green design, and ecological city development [27], integrating climate suitability into waterscape design to maximize the regulatory benefits is the key point to be considered in the current landscape design of campus square water bodies. Therefore, the main research of this study has been to quantify the scale, shape, and dispersion of water bodies, respectively, with the purpose of regulating the campus microclimate environment. The impact of different water body layout factors on the microclimate of a campus square is analyzed with the ENVI-met5.0.2 and Arcgis software tools. Then, the water body layout pattern that is suitable for the campus squares was defined according to the research results.

## 2. Materials and Methods

### 2.1. Overview of the Study Area

**Figure 1:**
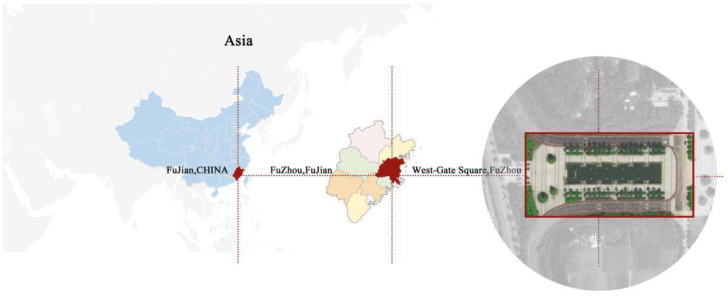
Location map of the study area.

Fuzhou City is located on the southeast coast of China, between 25°15′ N–26°39′ N and 118°08′ E–120°31′ E (Figure 1). It is known as “China’s furnace” due to the number of high temperature days above 35 °C in summer, which tops the list of cities in China [28]. Through field research and literature review, it has been found that the Fujian Agriculture and Forestry University has a large number of campus squares of various types, but they still fail to meet the needs of students and teachers in withstanding summer’s high temperatures [29]. In this study, the West Gate Square, which has the largest area and the highest utilization rate, is selected as the theoretical model. As the entrance and exit of the campus for students and teachers, West Gate Square is close to the urban arterial road and has a flat terrain with a large area. The total area of the square is 8400 m^2^ and the width of the road on both sides is 8 m. Due to the high temperatures in summer, few students and teachers stay and play here during daytime, and the number of people who play here increases gradually after 18:00. It has a simple and easily recognizable rectangular appearance with open views and no tall buildings blocking the perimeter. The internal landscape is symmetrically arranged, including the symmetrically arranged greenery planting and rectangular water bodies. West Gate Square is set as the basis of the theoretical model, which is operable for modeling in the ENVI-met software.

### 2.2. Quantitative Indicators of Water Bodies

The book *Landscape Ecology* mentions the close relationship between the spatial structure of patches and landscape patterns [30]. Zhang quantified the factors of water bodies based on the landscape pattern in ecology, which was used to explain the microclimate of urban water bodies [31]; Ding used the landscape index to model the relationship between the spatial structure of water bodies and the microclimate [32]. Therefore, based on a comprehensive review of relevant literature and field research, water body scale, dispersion, and shape were selected in this study and quantified analogically as the water body area index, the water body dispersion index, and the water body shape index by referring to the landscape index quantification method. The quantification equations are as follows.

1. Water body area index

The water body area index is the ratio of the water body area to the campus square area. It is used to quantify the water body scale in this study.

(1) $$S=\frac{S_{1}}{S_{2}}$$

*S* is the percentage of the water body area, *S*_1_ is the total area of the water body, and *S*_2_ is the total area of the square.

2. Water body dispersion index

The water body dispersion index is the sum of the distance (in meters) between each suffix in the landscape and its nearest neighbor divided by the total number of suffixes with neighbors. To compare the degree of water body dispersion between different scales with each other, this study further optimizes on the average proximity index. The formula added the total area factor of water bodies to quantify the dispersion degree of layout between different water bodies.

(2) $$FSD=\frac{\sum_{n=1}^{n}\sum_{j=1}^{n-1}h_{ij}}{n}$$

In this index, *i* refers to one of the suffix blocks, *j* refers to the remaining other suffix blocks, and *n* is the total number of suffix blocks.

(3) $$FSD^{*}=\frac{FSD}{\sqrt{\sum_{i=1}^{n}A}}$$

*FSD* refers to the average of nearest neighbor distance, *A* refers to the area of each water body, and *n* refers to the total number of water bodies.

3. Water body shape index

The quantitative formula for the water body shape index is a complex index that determines the shape of the suffix block based on its aspect ratio. According to the formula, the closer the *S* value is to 1, the more the shape tends to be square. The range of values: *XZ* ≥ 1, unlimited. It is used to quantify the complexity of the water body shape in the study.

(4) $$XZ=\frac{0.25P}{\sqrt{A}}$$

*P* is the perimeter of the water body and *A* is the area of the water body. (The reference shape is a square.)

### 2.3. Establishment of ENVI-Met Model

#### 2.3.1. Model Parameter Setting

According to the size of the simulated site, a model with a grid size of 190 m × 100 m × 100 m was built with ENVI-met. The grid cell size was set as dx = 2, dy = 2, and dz = 2. The climate of Fuzhou (26.08° N, 119.23° E) is maritime subtropical monsoonal, with the dominant summer wind being from the southeast. Soil, plants, wind speed, wind direction, and water bodies can interact with each other to produce interactive effects [31]. To prevent interaction effects from disturbing the results of this experiment, the wind speed was set as 2.5 m/s, the wind direction was set as 135° (southeast wind) [33,34], and the soil section and simple plant parameters were pre-set to fixed ratios in this study. Other parameters were set as shown in Table 1.

#### 2.3.2. Model Establishment

A theoretical model was established for West Gate Square of the Fujian Agriculture and Forestry University with the ENVI-met software. First, the area of the study area was calculated and converted into the number of grids which were needed in the software. Next, the data were converted to a bmp format file base map by Photoshop software, and then the theoretical model of the study area was created in the Space module of the ENVI-met software, saving the file type as the INX format.

Then, the simulation parameters were set for the mode. The main input parameters of the model included geographic information, meteorological conditions, soil conditions, and model output parameters [35]. A typical meteorological day of the summer solstice was selected as the starting time of the simulation, which was consistent with the monitoring time in the field. The simulated initial meteorological parameters were derived from the actual field measurement data and the Fuzhou City weather station data of that day (as shown in Table 1). The output parameters included air temperature, humidity, and wind speed. The simulated data from 8:00 to 18:00 was read from 5:00 to 19:00, and the output data was recorded for every 1 h interval. The saved file type was in the SIM format.

**Figure 2:**
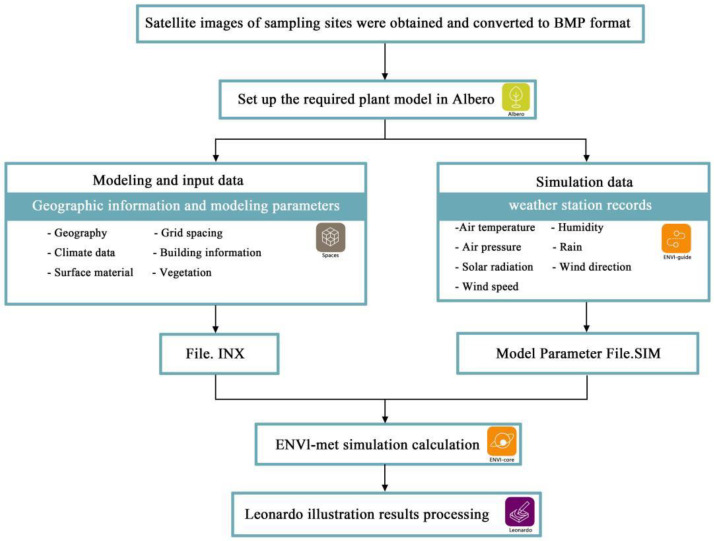
Sketch map of the modeling process.

Finally, the computer simulation was performed in the ENVI-core module. After the simulation, the results were visualized by the Leonardo module. The air temperature, humidity, and wind speed for each hour in the model were exported and all simulated data corresponding to the monitoring points were filtered from them. The data were compared with the actual measured data to verify the simulation accuracy. The model scheme was designed after the model testing standards are met. The detailed procedure is shown in Figure 2.

**Figure 3:**
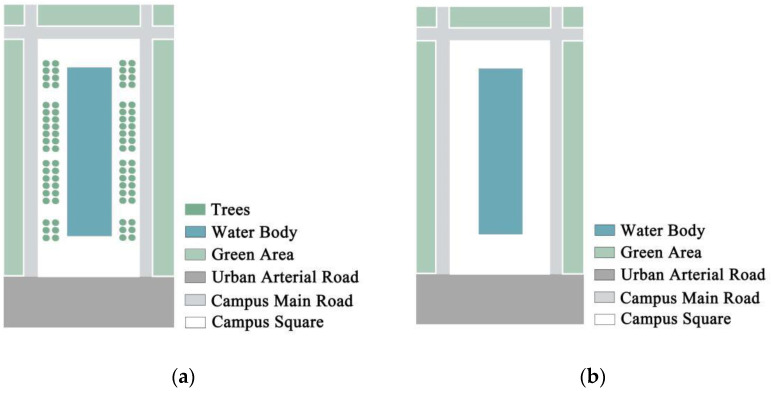
Theoretical model of the campus square: (**a**) with-tree model; (**b**) without-tree model.

To verify the influence of trees in this experiment, simulations were performed for the with-tree model and the without-tree model established by the Fujian Agriculture and Forestry University, respectively (as shown in Figure 3). The first simulation results indicated that the simulation data of both the with-tree model and the without-tree model were within the allowable error range and conformed to the real measurement results, which were both acceptable for the next experiment. To maximize the use of the square space and to avoid the situation where water bodies cannot be set up because of the existence of trees, the without-tree model was chosen for subsequent experiments. The West Gate Square area was selected and the without-tree model was verified again to ascertain whether it conformed to the real measurement data. The second simulation results indicated that the without-tree model of West Gate Square met the model simulation conditions [36] and could be used as the theoretical model for this experiment.

Square specifications: 140 m × 60 m.

Experimental area: 190 m × 100 m.

Road width: urban arterial road: 30 m; campus main road: 8 m.

Green area of plants on both sides: 12 m.

#### 2.3.3. Model Validity Verification

**Figure 4:**
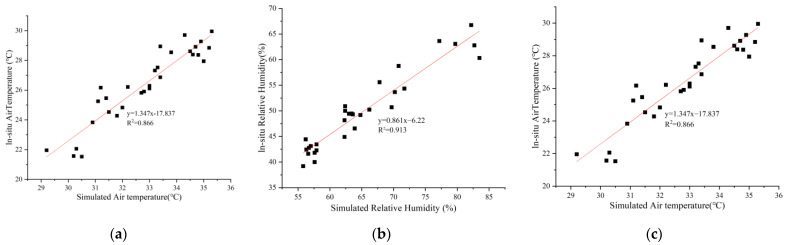
Correlation comparison between the measured and the simulated results: (**a**) Temperature; (**b**) Humidity; (**c**) Wind speed.

To improve the scientificity and accuracy of the microclimate simulation with the ENVI-met software, the monitoring points simulated by the software were set at the same locations as the actual test points. When comparing the simulated data results of the monitoring points with the actual measured test point data results in the field for calibration, if the error from simulated and measured results met the requirements of the simulation experiment, further experiments could be carried out [37]. The actual measurements were taken from 8:00 to 18:00 on 22 June 2022 at two-hour intervals. Temperature, humidity, and wind speed data were obtained simultaneously with a weather meter (kestrel 4500) at a distance of 1.4 m from the ground. Keeping the model substrate, road width, and square layout in the same way as the original site, the simulation time was set from 5:00. to 19:00, and the simulation data from 8:00 to 18:00 were read. The correlation analysis was performed between the measured and simulated results (Figure 4). The errors that resulted from the simulated and measured results met the simulation requirements [38], indicating that the ENVI-met software was suitable for this experiment and could reflect the microclimate change characteristics in the site.

#### 2.3.4. Model Schemes

**Figure 5:**
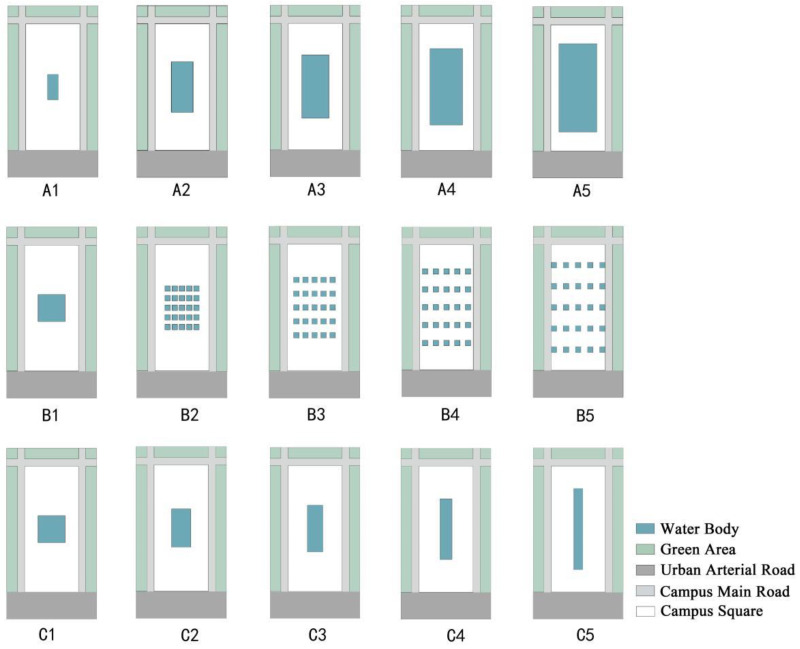
Illustration of 15 campus square water layout models. (**A1**–**A5**), different water body scale models. (**B1**–**B5**), different water body dispersion models. (**C1**–**C5**), different water body shape models.

By quantifying the scale, shape, and dispersion of water bodies, a single-factor experimental scheme was designed, and a total of 15 different water body layout model schemes were identified (Figure 5). Among them, five different area index (A) models of water bodies, five different dispersion index (B) models of water bodies, and five shape index (C) models of water bodies were included. One group of water body-free models numbered A0 was also set as the reference group. The detailed water body dimensions for each model scheme are shown in Table 2.

### 2.4. Data Analysis Methods

The data of temperature, humidity, and wind speed at each point of the site were obtained by the ENVI-met software simulations, and descriptive and correlation analyses of the temporal variation characteristics of the campus square microclimate were analyzed by origin 2021 and SPSS 26.0. Then, referring to the method of Wania et al. [39], the results of the campus square microclimate were imported into ArcGIS 10.8.1 for superposition analysis. The A0 data of the campus square under the condition of no water body was used as the original value, and the simulated values and the original values of different water body layout factors were superimposed; that is, the cooling (humidifying) effect of the same location = original value (simulated value)-simulated value (original value), which was used to obtain the global cooling and humidifying effect of the water body. Based on this, the Explore Data tool in ENVI-met was used to derive data at 2 m intervals of 40 m starting from the boundary of the water body in the downwind direction, and the data was exported for averaging, which was used to explore the influence range of the cooling and humidifying effects of different water body layout factors.

## 3. Results and Analysis

The simulation results of microclimate effects under different water body layouts were obtained by using the ENVI-met software. The spatial and temporal characteristics of the campus microclimate with different water body layout factors were analyzed from three indexes of temperature, humidity, and wind speed to clarify the relationship between water body layout factors and microclimate, and to determine the water body layout mode of the campus square.

### 3.1. Temporal Characteristics of the Microclimate with Different Water Layout Factors

#### 3.1.1. Temperature

**Figure 6:**
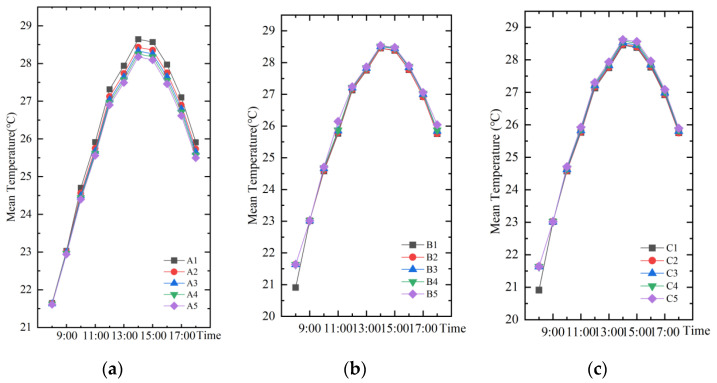
The trend of temporal mean temperature change for different water body layout factors: (**a**) Water body area index; (**b**) Water body dispersion index; (**c**) Water body shape index.

**Figure 7:**
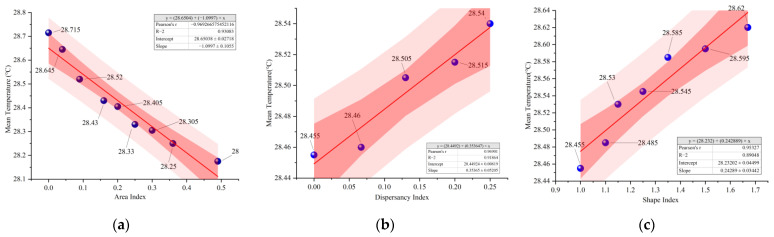
Correlation analysis of temperature and microclimate for different water layout factors: (**a**) Water body area index; (**b**) Water body dispersion index; (**c**) Water body shape index.

As shown in Figure 6, the trends of temporal mean temperature change for different water layout factors are the same, with the highest temperature at 14:00 and the lowest temperature at 8:00. The temperature at 14:00 was taken for correlation analysis with the three water layout factors (Figure 7). The relationship between the temperature and the change of area index (A) can be fitted as *y* = −1.1*x* + 28.65, and the relationship between the temperature and the change of water body dispersion index (B) can be fitted as *y* = 0.35*x* + 28.45. The relationship between the temperature and the change of shape index (C) of the water body can be fitted as *y* = 0.24*x* + 28.23. The results show that all three water body layout factors can significantly affect the change in air temperature; the water body area index and air temperature are negatively correlated; shape index, dispersion index and air temperature are positively correlated.

#### 3.1.2. Humidity

**Figure 8:**
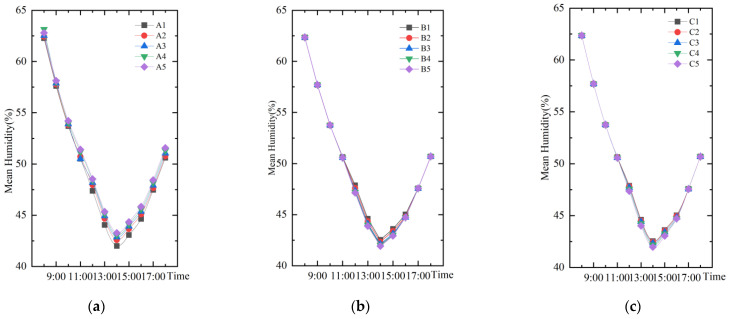
The trend of temporal mean humidity change for different water body layout factors: (**a**) Water body area index; (**b**) Water body dispersion index; (**c**) Water body shape index.

**Figure 9:**
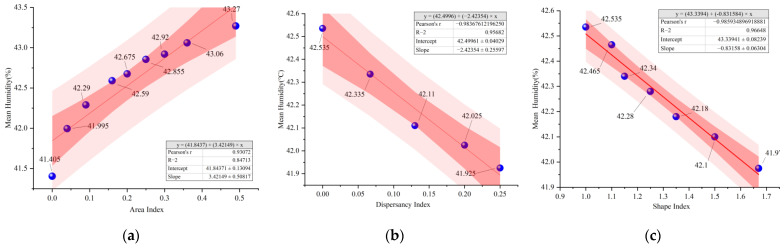
Correlation analysis of humidity and microclimate for different water layout factors: (**a**) Water body area index; (**b**) Water body dispersion index; (**c**) Water body shape index.

As shown in Figure 8, the trends of temporal mean humidity change for different water layout factors are the same, with the lowest humidity at 14:00 and the highest humidity at 8:00. The humidity at 14:00 was taken for correlation analysis with the three water layout factors (Figure 9). The relationship between humidity with the change of area index (A) can be fitted as *y* = 3.42*x* + 41.84 Humidity with the change of dispersion index (B) can be fitted as *y* = −2.42*x* + 42.5. Humidity with the change of shape index (C) of the water body can be fitted as *y* = −0.83*x* + 43.34. The results show that all three water body layout factors can significantly affect the humidity changes. The water body area index and humidity are positively correlated. However, the water shape index, the dispersion index and humidity are negatively correlated.

#### 3.1.3. Wind Speed

**Figure 10:**
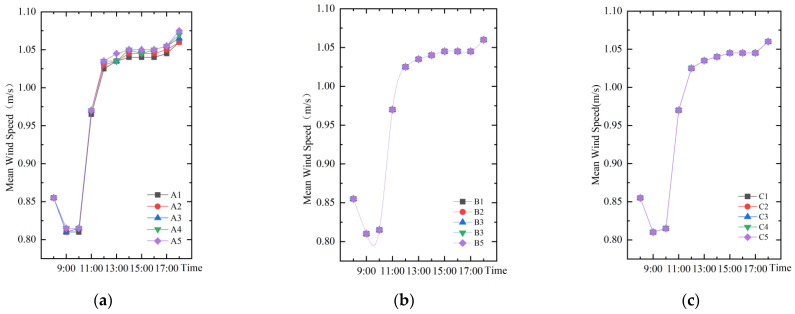
The trend of temporal mean wind speed change for different water body layout factors: (**a**) Water body area index; (**b**) Water body dispersion index; (**c**) Water body shape index.

**Figure 11:**
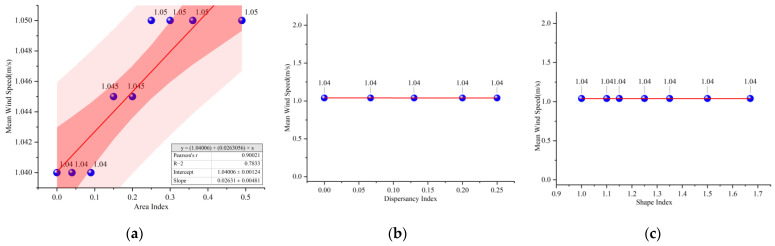
Correlation analysis of wind speed and microclimate for different water layout factors: (**a**) Water body area index; (**b**) Water body dispersion index; (**c**) Water body shape index.

As shown in Figure 10, the trends of temporal mean wind speed change for different water layout factors are the same. The wind speed at 14:00 was taken for correlation analysis with the three water layout factors (Figure 11). The relationship between square wind speed with the change of water body area index (A) can be fitted into the *y* = 0.03*x* + 1.04. However, the mean wind speed remains constant at *y* = 1.04 regardless of the variation of dispersion index (B) and shape index (C). The results show that the water body area is positively correlated with the wind speed, while the water body shape and dispersion of are not related to the wind speed.

### 3.2. Spatial Characteristics of the Microclimate with Different Water Layout Factors

#### 3.2.1. Cooling Effect

**Figure 12:**
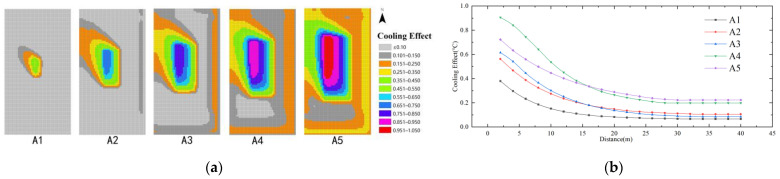
Cooling effect of water body area index: (**a**) Impact distribution map; (**b**) Impacts with distance.

As the wind direction of the model set is 135° in the southeast direction (summer wind), the affected areas of different water body layout model schemes are mainly concentrated in the downwind areas. As shown in Figure 12, the maximum cooling effect of the water body area index (A) is 1.05 °C, and the farthest influence distance is 30 m. Comparing the simulation schemes of different water body area indexes (A), it can be seen that the cooling effect of the water body gradually increases with the increasing area index. Until the area index increased to 0.36 (model A4), the cooling capacity of model A5 decreased instead of increasing. After a distance of 30 m from the water body, the difference in the cooling effect of the water body between A4 and A5 was small (<0.02). It can be seen that the cooling effect of simulation scheme A4 is the best.

**Figure 13:**
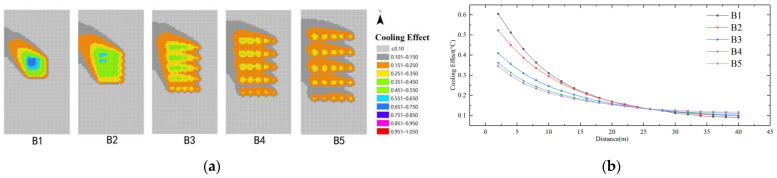
Cooling effect of the water body dispersion index: (**a**) Impact distribution map; (**b**) Impacts with distance.

As shown in Figure 13, in the case of the same distance, comparing the cooling effect of different water body dispersion indexes (B), the cooling effect of the model scheme is ranked as B1 > B2 > B3 > B4 > B5. The maximum cooling effect of the water body dispersion index (B) is 0.65 °C, and the farthest influence distance is 28 m. When the distance from the water body is >28 m, the difference in the cooling effect of the five schemes is very small (<0.04), which is negligible. It can be seen that the smaller the water body dispersion, the more obvious the cooling effect on the perimeter of the water body in the case of the area share of the water body is determined. Among them, the cooling effect of model scheme B1 is the best, and the best impact range is 0–28 m.

**Figure 14:**
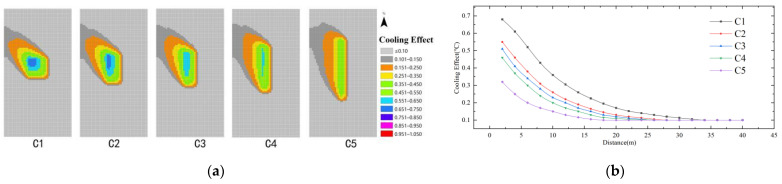
Cooling effect of the shape index of the water body: (**a**) Impact distribution map; (**b**) Impacts with distance.

As shown in Figure 14, the cooling effect of the model scheme at the same distance is C1 > C2 > C3 > C4 > C5. The minimum cooling value is 0.1 regardless of the variation of the shape index. The maximum cooling effect of the water body shape index (C) is 0.75 °C, and the farthest influence distance is 36 m. The cooling distance of model schemes C1–C5 are 36 m, 32 m, 28 m, 24 m, 20 m, and the cooling distance of C1 is the farthest. It can be seen that the more aggregated the water body shape, the better the cooling effect; in which case C1 has the best cooling effect and the longest distance, 36 m.

#### 3.2.2. Humidifying Effect

**Figure 15:**
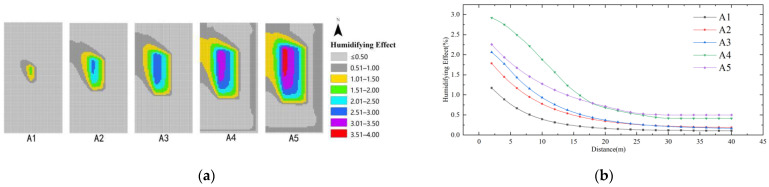
Humidifying effect of water body area index: (**a**) Impact distribution map; (**b**) Impacts with distance.

The humidifying effect of the water body layout model in summer is shown in Figure 15. The humidifying effect of the water body area index (A) is 4%, and the farthest influence distance is 30 m. Comparing the humidifying effect of different water bodies with area index (A), it can be seen that the humidifying effect of water bodies gradually increases with the increasing area index. Until the area index increases to 0.36 (scheme A4), the humidifying capacity of model scheme A5 decreases instead of increasing. After the distance of 30 m from the water body, the difference between the water humidifying effect of A4 and A5 is very small (<0.02). It can be seen that A4 has the best cooling effect.

**Figure 16:**
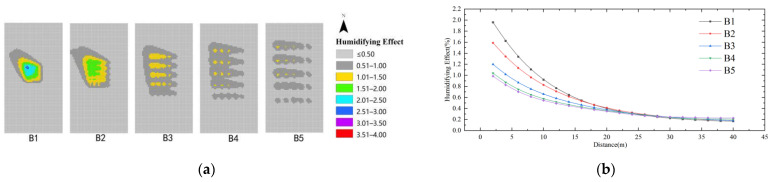
Humidifying effect of water body dispersion index: (**a**) Impact distribution map; (**b**) Impacts with distance.

As shown in Figure 16, in the case of the same distance, comparing the humidifying effect of different water dispersion indexes (B), it is seen that the humidifying effect of the water dispersion index is ranked as B1 > B2 > B3 > B4 > B5. The best humidifying effect of the water body dispersion index (B) is 3%, and the farthest influence distance is 28 m. The humidifying effect is not obvious when the distance from the water body is >28 m. It can be seen that the smaller the water body dispersion, the better the humidifying effect is on the environment around the water body when the area share of the water body is determined. Among them, B1 has the strongest humidifying effect, and the best influence range is 0–28 m.

**Figure 17:**
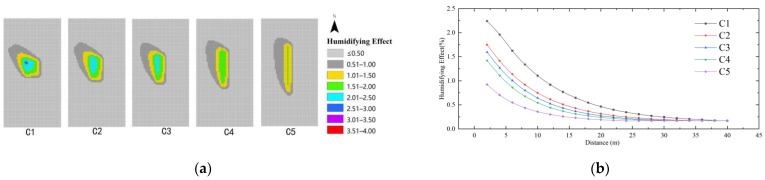
Humidifying effect of shape index of the water body: (**a**) Impact distribution map; (**b**) Impacts with distance.

As shown in Figure 17, in the case of the same distance, the humidifying effect of the model scheme is ranked as C1 > C2 > C3 > C4 > C5. The best humidifying effect of the shape index (C) is 2.8%, and the farthest influence distance is 36 m. Regardless of how the shape index changes, the minimum humidifying value is 0.17. The humidifying distances of C1–C5 are 36 m, 32 m, 28 m, 24 m, and 20 m, respectively. This indicates that the more aggregated the water body shape, the better the humidifying effect and the farther the influence distance. Among them, C1 has the strongest humidifying effect and the farthest influence distance with 36 m.

### 3.3. Determination of the Water Body Layout Mode of the Campus Square

The water body layout mode is closely related to the microclimate effect, and a reasonable combination of water body layout factors can effectively improve the campus microclimate environment. The experimental results show that the cooling and humidifying effect of model schemes B1 and C1 is the best under the condition of a certain water body area. In other words, the cooling and humidifying effect of the water body layout method with shape index XZ = 1 and dispersion index FSD = 0 is the best. The microclimate effect of model scheme A4 is the best for a certain water body dispersion and shape. It means that the cooling and humidifying effect of water body area S = 0.36 is the best. The optimal solution of all factors was integrated to derive the best microclimate-based water layout mode for the campus square: water body area index S = 0.36, water body dispersion FSD = 0, and water body shape index XZ = 1.

Based on the common campus square size of 35 universities in Fuzhou [40], this study brings in the optimal campus square water layout mode to explore the optimum square boundary ratio under this mode so as to guide the campus square practice better. Five schemes with square boundary ratios of 1:1, 1:2, 2:3, 3:5, and 4:5, named S1–S5, with a uniform area of 8400 m^2^, were established for the experiments. The parameters were set as Table 1 as before.

**Figure 18:**
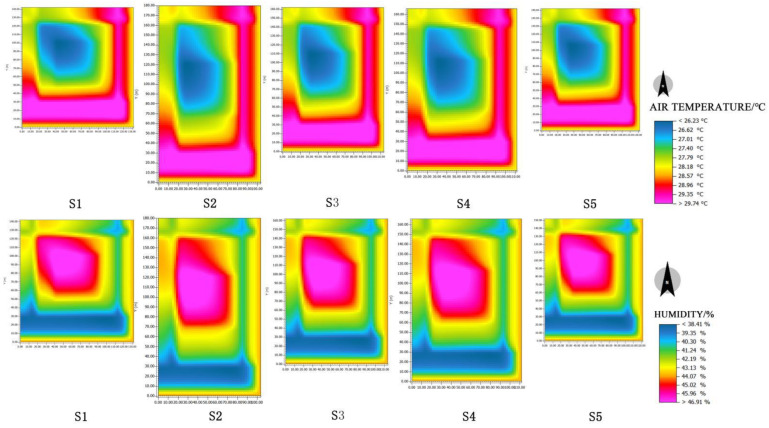
Temperature and humidity plane distribution of each square scheme.

**Figure 19:**
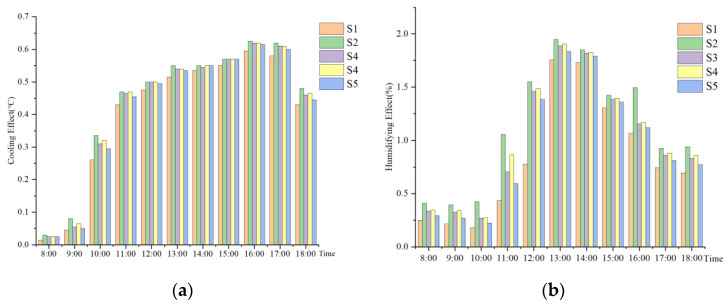
Cooling and humidifying effect of each square scheme: (**a**) Cooling effect; (**b**) Humidifying effect.

As shown in Figure 18, the temperature and humidity of different squares (S) are ranked as follows: S2 > S4 > S3 > S5 > S1. Under a certain condition of water body layout, comparing the cooling and humidifying effects of five schemes with the experimental reference group of no water body A0, as shown in Figure 19. The cooling and humidifying effect of scheme S2 is the strongest, with the maximum cooling value of 0.625 and the maximum humidifying value of 1.95. The next one is Scheme S4. Therefore, it can be concluded that the setting of the square boundary ratio at 1:2 can maximize the microclimate regulation effect under the certain condition of water body layout.

## 4. Discussion

The water body scale is the main layout factor that directly affects the microclimate effect, according to the analysis of the microclimate effect of different water body layout factor modeling schemes. Due to the large area of the water body, the water surface has high reflectivity and evaporation, which can remove some of the heat during air exchange, resulting in a good cooling and humidifying effect. Simultaneously, the open water surface expands the exchange with the dominant wind, which increases wind speed. This supports the findings of Saaroni, Jin, H [14,41], and others. It is shown that the larger the area of the water body, the further the distance of influence on the surrounding environment of the water body. However, when the area of water bodies exceeds 36%, the regulating effect of the microclimate decreases instead of increasing. At the same time, oversized water bodies prolong the time required for pedestrian passage and reduce human comfort. It is obvious that a larger scale of water body does not mean a better microclimate regulation effect. Second, the dispersion and shape of the water body can affect the cooling and humidifying of the square, but not wind speed. The greater the water body dispersion index, the smaller the difference in temperature and humidity in the square, and the greater the distance of the influence. This is consistent with Ding and Offerle’s findings [23,32]. The higher the aggregated water body dispersion index, the greater the cooling effect, which contradicts the findings of Xuan et al. [42]. It is presumed that the regulation effect is affected because of the different scales of the study area, the limited water body dispersion, and the percentage of dispersion area. Regarding water body shape, the more aggregated the water body shape is the squarer the shape tends to be. In addition, the better the potential for cooling and humidifying, and the farther the influence distance. This is consistent with the findings of Wang et al. [43]. In addition, it was found that the distribution state of temperature and humidity within the site is affected by the wind direction, resulting in an asymmetrical shape of the water body that affects the surrounding environment. The prevailing wind direction determines the direction of air flow. The air flow passing from a water body and carrying water vapor causes a decrease in temperature and an increase in humidity in the downwind area. Therefore, the cooling and humidifying effect is stronger in the downwind direction and weaker in the upwind direction with a single wind direction (southeast wind at 135°) and constant water body layout factors. It is consistent with the findings of Sen [13], Wang [44], and Mak et al. [45].

## 5. Conclusions and Prospect

To explore the relationship between the water layout mode and the campus microclimate, this study selected the West Gate Square of the Fujian Agriculture and Forestry University as the research object. The research content of the campus microclimate is enriched from the perspective of landscape quantification. The following conclusions are drawn.

(1) The scale, shape and dispersion of water bodies had significant effects on the temperature and humidity of the campus square. The water body scale was positively correlated with temperature and negatively correlated with humidity; the water body shape and dispersion were negatively correlated with temperature and positively correlated with humidity. All three factors had no significant effects on wind speed.
(2) From the perspective of the cooling and humidifying effect, the ranking of the regulating ability of water body layout factors is scale > shape > dispersion; the ranking of the influence range is shape (36 m) > scale (30 m) > dispersion (28 m).
(3) Based on the analysis of the three factors of water body scale, shape, and dispersion, the optimum layout mode of the water body with a certain area of the campus square is as follows. The area of the water body accounts for 36% of the total area of the square, and the shape is square, centralized and non-dispersive. The optimum layout mode of the campus square boundary is length:width = 1:2 under the circumstances that the layout factors of the water body are determined.

Suitable waterscape design can improve the square microclimate environment in the hot summer to some extent. The findings of this study can be generally applied in future open-space design. According to the research results, designing the active space in the downwind direction in the water body area can effectively improve human comfort. Integrating the water layout factors and other landscape factors can create a cooler public square space. Square water bodies should be designed with a reasonable increase in water area to avoid creating too large a water body area where the cooling and humidifying effect becomes worse. Furthermore, the layout of water bodies in campus squares is significant for other types of squares. For the entrance and the monumental square types, a large area and gathering water bodies can not only achieve a cooling effect, but also show the sense of order and solemnity of the square environment. For urban entertainment squares, commercial squares and other recreational natures, the use of a dispersed water arrangement can effectively alleviate the urban thermal environment and create a healthy climate. For example, a music dry-spray square and a fountain square (dispersion interval kept within 28 m).

In conclusion, this study constructed a series of water body layout models with different scales for three water body layout factors. Then, the models were simulated and calculated with the campus square and peripheral environment models. The optimum campus water layout mode based on the microclimate effect was determined as a result. This provides a theoretical basis for the future practice of campus squares and other squares.

Other attributes of water bodies should be considered in future research, such as the “microclimate effects of static and dynamic water” or the “wind speed and wind direction on water microclimate effect and ripple range as major influencing factors”. Meanwhile, other landscape factors in squares should be integrated into the research systems, such as plants, structures, and substrates to build a more complete theoretical model. In addition, the microclimate effect of water bodies in spring, autumn, and winter should be considered. This would mean that the water body design could be applied in every season and could provide a more complete theoretical reference and data support for the construction of campus landscape space.

## Figures and Tables

**Table 1:** Parameter settings of ENVI-met.

Parameter Name	Parameter Name	Parameter Values
Grid Settings	Model dimensions /Size of grid cell in meter	190 × 100 × 100/2 m × 2 m × 2 m
Model Location	Base settings	West Gate Square of Fujian Agriculture and Forestry University 26.08° N, 119.23° E
	Microscale roughness length of surface (m)	0.01
Time and date	Start date	22 June 2022
	Start time	5:00 am
	Total simulation time	14
Meteorological data	Specific humidity in 2500 m (g/kg)	7
	Wind direction	135 degrees (south-east)
	Windspeed (m/s)	2.5
	temperature range	17–28
Soil Section	Upper layer (0–20 cm)	65 ℃/50% RH
	Middle layer (20–50 cm)	70 ℃/50% RH
	Deep layer (50–200 cm)	75 ℃/50% RH
Simple plant parameters	Surface Albedo	0.20
	Foliage shortwave transmittance	0.30
	Height (m)	0.25
Soils and Surface	Campus road/ Urban arterial road	Asphalt road
	Campus square	Concrete pavement road
	Campus greenery	Grass 25 cm aver, dense

**Table 2:** Detailed dimensions of the campus square water layout model.

Influencing Factors	Parameter Name	Model Information				
Scale of the water body	Model number	A1	A2	A3	A4	A5
	Area Index (A)	0.04	0.16	0.25	0.36	0.49
	Water body size (m)	12 × 28	24 × 56	30 × 70	36 × 84	42 × 98
Dispersion of the water body	Model number	B1	B2	B3	B4	B5
	Dispersion index (B)	0.00	0.07	0.13	0.20	0.25
	Water body size (m)	30 × 30	21 × 42	17 × 52	15 × 60	10 × 90
Shape of the water body	Model number	C1	C2	C3	C4	C5
	Shape Index (C)	1.00	1.10	1.15	1.35	1.67
	Water body size (m)	30 × 30	30 × 30	30 × 30	30 × 30	30 × 30

## Data Availability

All images in the text were drawn by the author. The data used to support the findings of this study are available from the corresponding author upon request.
